# Acetylcholinesterase Regulates Skeletal *In Ovo* Development of Chicken Limbs by ACh-Dependent and -Independent Mechanisms

**DOI:** 10.1371/journal.pone.0161675

**Published:** 2016-08-30

**Authors:** Janine Spieker, Anica Ackermann, Anika Salfelder, Astrid Vogel-Höpker, Paul G. Layer

**Affiliations:** Developmental Biology and Neurogenetics, Technische Universität Darmstadt, Schnittspahnstrasse 13, D-64287, Darmstadt, Germany; Weizmann Institute of Science, ISRAEL

## Abstract

Formation of the vertebrate limb presents an excellent model to analyze a *non-neuronal cholinergic system* (NNCS). Here, we first analyzed the expression of acetylcholinesterase (AChE) by IHC and of choline acetyltransferase (ChAT) by ISH in developing embryonic chicken limbs (stages HH17-37). AChE outlined formation of bones, being strongest at their distal tips, and later also marked areas of cell death. At onset, AChE and ChAT were elevated in two organizing centers of the limb anlage, the *apical ectodermal ridge* (AER) and *zone of polarizing activity* (ZPA), respectively. Thereby ChAT was expressed shortly after AChE, thus strongly supporting a leading role of AChE in limb formation. Then, we conducted loss-of-function studies via unilateral implantation of beads into chicken limb anlagen, which were soaked in cholinergic components. After varying periods, the formation of cartilage matrix and of mineralizing bones was followed by Alcian blue (AB) and Alizarin red (AR) stainings, respectively. Both acetylcholine (ACh)- and ChAT-soaked beads accelerated bone formation *in ovo*. Notably, inhibition of AChE by BW284c51, or by the monoclonal antibody MAB304 delayed cartilage formation. Since bead inhibition of BChE was mostly ineffective, an ACh-independent action during BW284c51 and MAB304 inhibition was indicated, which possibly could be due to an enzymatic side activity of AChE. In conclusion, skeletogenesis in chick is regulated by an ACh-dependent cholinergic system, but to some extent also by an ACh-independent aspect of the AChE protein.

## Introduction

Acetylcholine (ACh) and its cholinergic components, e.g. cholinesterases (ChEs), choline acetyltransferase (ChAT), receptors (AChRs) and vesicular transporters (vAChT) are expressed not only at synapses, but in many non-neural cell types, suggesting synapse-independent roles of cholinergic systems (also known as *non-neuronal cholinergic systems*, NNCS), e.g. regulating development or immune responses [1–6]. In particular, roles of ChEs, e.g. acetyl- (AChE) and butyrylcholinesterase (BChE) in developmental processes should not be underestimated, since they represent—due to their high turnover rate—rate-limiting components within classical cholinergic signaling. Moreover, AChE presents a amidase side activity, which has been associated with developmental processes (Boopathy and Layer, 2004, Chinnadurai et al., 2015). Notably, ChEs cannot only function enzymatically by degrading acetylcholine (ACh), but may also act non-enzymatically, e.g. in neurite growth mechanisms [7, 8]. Often, a combination of several mechanisms may be in action [9]. Non-enzymatic actions of ChEs in cell-cell adhesion are supported by various findings, e.g. ChEs belong to the family of ChE-domain proteins [10], the existence of collagen-tailed forms of AChE and interaction of AChE with components of the ECM, like collagens or laminin [11, 12]. AChE not only can promote differentiation of particular cell types [13, 14], but sometimes AChE is associated with the process of apoptosis [15, further see Discussion]. Notably, non-neuronal actions of ChEs may have high relevance for various diseases [13, 16, 17].

Skeletal development in all vertebrates including human follows similar cellular and molecular mechanisms. Particularly, development of long bones in limbs by endochondral ossification represents an outstanding model to analyze NNCS, since interference by neural components is minimal (at least during its initial stages). Indeed, multiple reports exist on cholinergic actions in skeletogenesis [18, 19, further in Discussion]. For instance, nicotine applied *in vitro* supported proliferation and inhibited hypertrophic matrix formation of chondrocytes, delaying skeletal growth [18, 19]. Furthermore, AChE functions in development of the skeleton [3, 9, 13, 20, 21]. Molecularly, the skeletogenic master regulator Runx2 possesses an AChE promoter binding site [22], suggesting that by activating AChE Runx2 could counteract cholinergic actions during chondrogenesis, e.g. stimulate bone differentiation. Reports on promotion of apoptosis by AChE further nurtured interests in ChE functioning in regulation of developmental processes [15, 23–26]. Our earlier report on distinct and mutually exclusive expression patterns of both ChEs during limb development [27], and findings of a severely affected skeleton in an AChE/BChE double knockout mouse [9, 14] raised further questions about cholinergic functions in vertebrate skeletal development, such as follow: which cholinergic components are causally involved in chondrogenesis and/or in ossification? Are actions of AChE exclusively dependent on its ACh-degrading capacity?

Here, we took advantage of the chicken embryo as an easily accessible model system. First, we focused on ChE and ChAT expression patterns in chicken limbs, by using histochemical and ISH procedures on whole-mounted specimen, respectively. Then, we performed loss-of-function *in ovo* experiments by implanting into one limb bud beads pre-soaked either with i) ACh, or ii) ChAT protein, iii) with the AChE inhibitor BW284c51, and iv) with the antibody MAB304. The latter two agents inhibit AChE activity, but could also affect AChE´s enzymatic side or adhesive actions [8, 28, 29, 30]. The effects on chondrogenesis and ossification were analyzed by Alcian blue (AB) and Alizarin red (AR) stainings, respectively. Our findings support the notion that a non-neuronal cholinergic system (NNCS) is involved in skeletal development of chicken limbs, to which AChE contributes both by an ACh-dependent and an ACh-independent mechanism.

## Materials and Methods

### Chick embryos

Fertilized chicken eggs from *White Leghorn* (LSL hatchery, Dieburg, Germany) were incubated at 37°C and 60–65% humidity, until they had reached the desired stages (stage 17 to 37), according to Hamburger and Hamilton [31].

Ethics statement: According to German animal welfare regulations ("Deutsches Tierschutzgesetz"), chicken embryos until hatching are not assigned the legal status of "animals"; therefore approval of an ethics committee was not required for this study.

### In vivo bead implantations of the developing chick embryo

The eggs were windowed and embryonic membranes removed by using sharpened tungsten needles. One agarose bead (Affi-Gel Blue Gel Beads, Biorad Laboratories, Munich, Germany) soaked for at least 2h in the respective agent (10 mM of ACh, or BW284c51, or in 100 μg/ml of purified ChAT protein, or in 1 mg/ml MAB304 (clone AE-2, purchased from Chemicon Co., EMD Millipore) [8, 32, 33–37] was transferred with a fine forceps onto the embryo, and—with the aid of a fine needle—positioned into one of either front or hind limb anlage of staged HH17-22 embryos. In separate control experiments, beads soaked in PBS attested that bead treatment alone had no adverse effects on limb development. Due to the small size of limb buds, placement of beads varied (for bead placements, see Tables 1–4). The eggs were then sealed and left (without turning) to develop at 38°C/65% humidity until they had reached the desired stage. Embryos were fixed in 4% paraformaldehyde in PBS at 4°C for 2 to 48 hours.

**Table 1:** ACh bead implantation.

No. expt.	treatment period	bead posit. at implantat.	bead at fixation	treated limb	staining	remarks
V46	HH25-30	near tip of bud, slightly post	visible above middle foot bone	right leg (rl)	Alcian blue & Alizarin red (AB/AR)	treated leg enlarged
V70	HH23-28.5	1/2 inside of bud tip,	clearly visible	left wing (lw)	AB/AR	treated wing enlarged
V101	HH18-38	on posterior	detectable at feather bases	right wing (rw)	AB/AR	mineralisation in radius of treated wing accelerated
V123	HH18-30	center of bud	not detectable	rw	AB/AR	chondrogenesis in treated wing accelerated
V148	HH25-27.5	1/4 inside of tip of bud	easily detectable	lw	AB/AR	treated wing slightly enlarged
V152	HH25-32	near tip of bud	not detectable	lw	AB/AR	treated wing enlarged
V184	HH17.5–37	center of bud	not detectable	rw	AB/AR	radius of treated wing slightly more mineralized
V244	HH17.5–37	center of bud	detectable at bone head of radius	rw	AB/AR	mineralization of radius and ulna of treated wing stronger than in contralateral wing
V248	HH18-38	center of bud, slightly post	clearly visible below radius	lw	AB/AR	mineralization of treated wing clearly stronger

total 60, dead 12, with effect 9, w/o effect 39

**Table 2:** ChAT bead implantation.

No. expt.	treatment period	bead posit. at implant.	bead at fixation	treated limb	staining	Remarks
V86	HH18-36	center of bud	not found (nf)	right wing (rw)	Alcian blue & Alizarin red (AB/AR)	mineralisation in Ulna stronger area more expanded
V162	HH18.5–37	center of bud,slightly post	found in joint of humerus	rw	AB/AR	mineralisation significantly accelerated
V185	HH18-37	center of bud	nf	rw	AB/AR	mineralisation of digit bones
V187	HH17.5–38	center of bud	found at feather base	rw	AB/AR	mineralisation accelerated
V193	HH18-38	center of bud	found below head of ulna	rw	AB/AR	minor differences of mineralisation
V195	HH18.5–39	center of bud	found in hand joints	rw	AB/AR	mineralisation accelerated
V196	HH18-32	center of bud	found in hand joints	left wing (lw)	AB/AR	chondrogenesis accelerated wing appears enlarged
V197	HH17.5–37.5	center of bud	nf	lw	AB/AR	mineralisation slightly accelerated
V213	HH17.5–24.5	center of bud	found in ulna	rw	AB/AR	chondrogenesis and limb growth significantly accelerated
V271	HH23-27	1/3 inside tip of bud	found	rw	Karnovsky-Roots (AChE)	no effect on AChE activity

total 65, dead 19, with effect 10, w/o effect 36

**Table 3:** BW284c51 bead implantation.

No. expt.	Treatment period	bead posit. at implant	bead at fixation	treated limb	staining	remarks
V30	HH25.5–32	far tip of limb	detectable between digits	right wing (rw)	Alc blue & Aliz. red (AB/AR)	treated wing smaller than contralaterally
V38	HH26-32	near tip of limb	detectable between digits	right leg (rl)	Karnovsky Roots (AChE)	AChE activity lost in treated area
V40	HH17-21	center of bud	clearly visible	left wing (lw)	K&R (AChE)	AChE activity strongly decreased on treated side
V48	HH26-29.5	near tip of limb	detectable between digits	rl	K&R (AChE)	AChE activity lost in treated area
V52	HH25.5–28	1/3 inside of tip of limb	detectable	rl	K&R (AChE)	treated leg: much less muscle fibers
V118	HH27-37	center of limb	not found	lw	AB/AR	treated wing: less mineralization
V163	HH17-36.5	center of limb bud	not found	lw	AB/AR	treated wing: slightly less mineralization
V167	HH18-36.5	center of limb bud	not found	rw	AB/AR	treated wing presents loss of mineralization
V201	HH18-36	center of bud, slightly posterior	detectable below hand joints	rw	AB/AR	mineralization delayed in treated wing, esp. in ulna
V208	HH17.5–26	center of bud, slightly posterior	detectable	lw	AB/AR	formation of cartilage slightly delayed
V285	HH18-36	center of bud	not found	rw	AB/AR	mineralization slightly delayed in ulna

total 55, dead 8, with effect 11, w/o effect 36

**Table 4:** MAB304 bead implantation.

No. expt.	treatment period	bead posit. at implant	bead at fixation	treated limb	staining	remarks
AK5	HH17-25.5	center of bud	detectable	left wing (lw)	Alcian blue & Alizarin red (AB/AR)	cartilage formation delayed
AK10	HH17-37	center of bud, slightly post	not found	right wing (rw)	AB/AR	mineralization strongly delayed
AK11	HH18-37	center of bud	not found	rw	AB/AR	mineralization strongly delayed
AK13	HH18-36.5	center of bud	not found	rw	AB/AR	mineralization clearly delayed
AK14	HH17.5–34.5	center of bud	not found	lw	AB/AR	strange deformations of dorsal vertebrae
AK22	HH17-36	center of bud	not found	lw	AB/AR	mineralization slightly delayed
AK23	HH18-36	center of bud	not found	rw	AB/AR	mineralization clearly delayed
V91	HH18.5–30	center of bud, slightly post	detectable	right leg (rl)	Karnovsky Roots (AChE)	no effect on AChE activity
V104	HH18-30.5	center of bud	detectable	rl	AB/AR	no effect on AChE activity
V170	HH17.5–37	center of bud	not found	rw	AB/AR	mineralization clearly delayed
V172	HH17.5–37	center of bud, slightly post	detectable in shoulder	lw	AB/AR	mineralization delayed
V173	HH17-38	center of bud	in joint below humerus	rw	AB/AR	no mineralization in radius, addit. growth of small digit
V263	HH18-31	center of bud	detectable	rl	K&R (AChE)	no effect on AChE activity
V268	HH 18	center of bud	detectable	rw	Karnovsky Roots (AChE)	no effect on AChE activity

total 25, dead 4, with effect 14, w/o effect 7

### Alcian blue and/or Alizarin red stainings

For Alcian blue/Alizarin red skeletal stainings, whole embryos or single limbs were harvested and fixed in 4% paraformaldehyde at 4°C overnight. The embryos or limbs were stained in Alcian blue solution (0.1 mg Alcian blue in 2% acetic acid/EtOH) overnight. After several hours in 95%, 70%, 40% und 15% ethanol, they were transferred to a 1% trypsin solution for 2 h to digest and clear the tissue. After staining in Alizarin red S solution for 16 h (40 mg/l Alizarin red in 0.5% KOH), skeletons were cleared in a series of 0.5% KOH/25% glycerol, 0.5% KOH/50% glycerol and stored in 0.5% KOH/70% glycerol at 4°C.

### In Situ Hybridization for ChAT in whole mounted embryos

Embryos to be used for whole-mount in situ hybridization (ISH) were harvested, fixed at the desired stage in 4% paraformaldehyde in PBS overnight, dehydrated and stored in 100% methanol. ISH for choline acetyltransferase (ChAT) of whole-mounted chicken embryos with digoxigenin-labeled chicken antisense RNA probes was performed as described previously (ChAT; T7 polymerase; EcoRI-linearized pBsSK, see [38–40]). Briefly, dehydrated embryos were rehydrated through steps of decreasing methanol concentrations (75, 50, 25%) in PBS, to be washed in PBS-0.1% Tween20 at RT. At RT, they were treated with proteinase K (10 μg/ml), washed twice in PBS-0.1% Tween20 and fixed for 20 min in 4% paraformaldehyde at RT. Then they were incubated for 5 min at RT and then 60 min at 68°C in pre-hybridization buffer (25 ml formamide, 12.5 ml 20x SSC, pH 4.5, 50 μl of 50 mg/ml heparin stock solution, 250 μl 10% yeast RNA, 5 ml 10% SDS, water up to 50 ml). Then 30 μl/ml of DIG-labelled probe was added and left overnight for hybridization. Antibody binding: 4x wash in pre-hybridization buffer at 68°C (2x 5 min, 2x 30 min); at 68°C wash for 10 min in 1.5 ml pre-hybrid buffer, mixed 1:1 with 1xMABT (5x stock solution, 11.6 g maleic acid, 8.7 g NaCl, 11 g Tween20 in 1000 ml). After 3 washes in 1xMABT at RT, transfer into MABT plus 2% blocking reagent (BR, Roche, REF 11096176001) for 1 h; and then same again including 20% heat-inactivated sheep serum followed. Then incubation with a sheep polyclonal anti-DIG-antibody (Roche, REF 11093274910) in a 1:1000 of previous buffer at 4°C overnight, followed by slight stirring. Then, the antibody solution was replaced by three washes in 1xMABT at RT. Embryos were washed at least thrice with NTMT buffer (1ml 5M NaCl, 5ml 1M Tris-HCl, pH 9.5, 2.5 mL 1M MgCl_2_, 5 ml Tween20, water up to 50ml) to adjust pH to 9.5. Staining solutions consisted of 4.5 μl NBT and 3.5 μl BCIP in 1ml NTMT buffer. Embryos were covered with staining solution and incubated light-tight at RT and slightly stirring, until a dark blue precipitation occurred. Stained embryos were washed several times in 1xPBS at RT to stop staining reaction and stored at 4°C.

### Cholinesterase stainings

Acetylcholinesterase staining in whole-mounted embryos was performed using the Karnovsky and Roots technique [41], and was performed as follows: after fixation, embryos were treated for 12 h in PBS containing 0.5% Triton-X-100, followed by a 12 h wash in Tris-maleate buffer including 1% Triton-X-100, and then a 2 h wash in Tris-maleate alone. Then embryos were incubated in a solution consisting of 32,5 ml of 0,1 M Tris-maleate, pH 6.0, 2,5 ml of 0.1 M sodium citrate, 5 ml of 30 mM copper sulfate, 5 ml of 5 mM potassium hexacyanoferrate III, 0,5 ml of 1% Triton-X-100, 50 μl of 10 mg/ml proteinase K, and 4 ml dest. water, including 36 mg of ATC and 0,5 ml of 10 mM iso-OMPA (inhibits BChE) for AChE activity, or 50 mg BTC plus 0,25 ml of 10 mM BW284c51 for BChE activity, respectively. For Karnovsky-Roots stainings of cryosections, treatment with Triton-X-100 was omitted. In brief, frozen cryosections were dried on a heating plate and pretreated in Tris-maleate buffer for 20 minutes. Then slides were incubated in staining solution (all doses as above) at 37°C for up to 4 hrs for AChE; for BChE, reaction lasted up to 10 hrs. Reactions were stopped by washing of slides in PBS, which then were covered by Kaisers glycerol.

### Statistics

For histochemistry and ISH all experiments were performed at least threefold; representative figures are presented. For in vivo bead implantations, all experiments were performed manyfold. Each one was documented separately (incl. specific location of bead) and is reported in Table 1.

## Results

### 1. AChE as a leading cholinergic formative component in chicken limbs

#### AChE precedes and opposes ChAT in early limb buds

**Fig 1:**
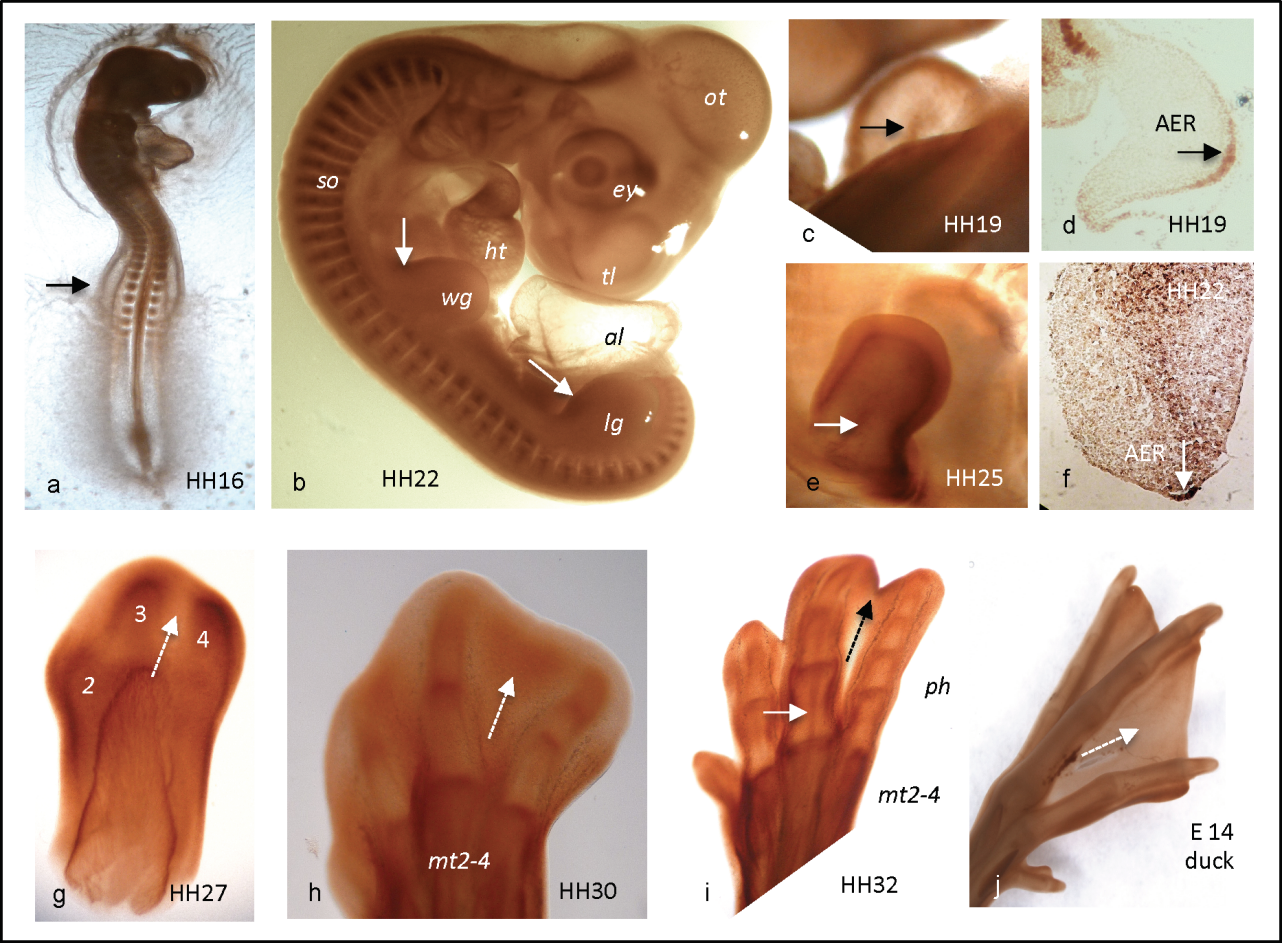
**AChE activity in limbs of whole-mounted chick (a-i) and duck (j) embryos.** a) weak AChE in HH16 hind limb bud (arrow); b) AChE in HH22 embryo. Limb buds have extended distally; note AChE is strongest at their rostral corners (arrows). c) pair of hind limb buds at HH19. In ventral aspect (upper), AChE is strong at rim and along a longitudinal centre stripe (arrow); AChE is higher on dorsal (lower limb) than on ventral side (upper); d) stained cryosection at HH19; note internal AChE^+^ cells, and ectodermal AChE, being strongest at AER (arrow); e) pair of front limb buds at HH25; note outer rim and inner stripe of AChE activity (arrow); f) AChE on cryosection at HH22; note AChE at AER; g) at HH27, AChE outlines ends of future digits 2–4 ("2–4"); note interdigital space is free of AChE (stippled arrow); h) by HH30, foot structure has become complex, indicating individual phalanges; note that interdigital areas begin to present AChE (stippled arrow); i) foot by HH32 presents four digits, individual phalanges clearly outlined by AChE; note interdigital space shows AChE at rim (stippled arrow); j) AChE expression in foot of E14 duck embryo; note interdigital space is free of AChE (stippled arrow). al, allantois; dt, digit; ey, eye; lg, leg; ht, heart; mt, metatarsus; ot, optic tectum; ph, phalange; so, somites; tl, telencephalon; wg, wing.

**Fig 2:**
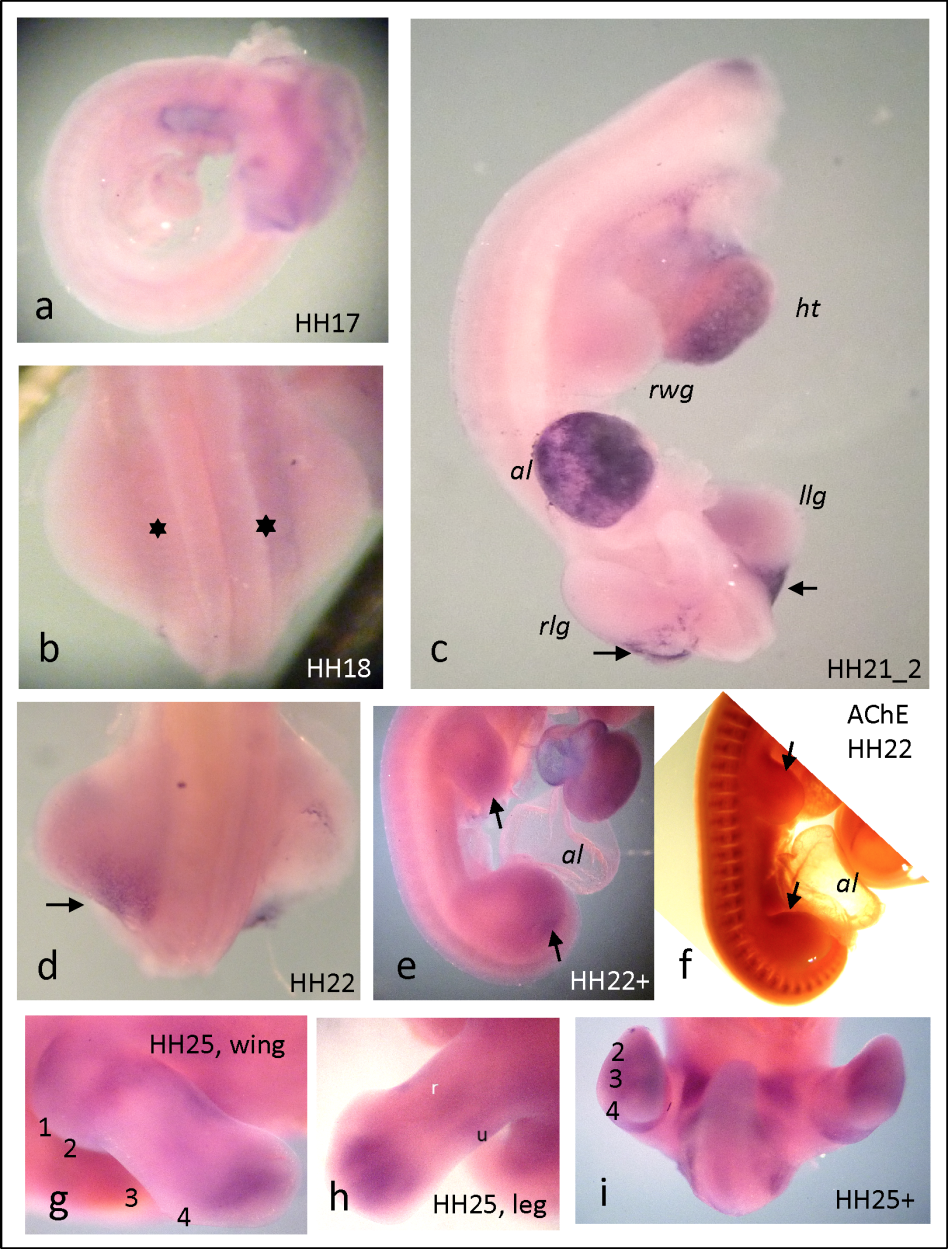
ChAT expression by ISH in whole-mounted embryos from HH17 to HH25. a) onset of ChAT expression in head and heart; b) first ChAT expression at proximal basis of HH18 hindlimb; c) ChAT in HH 21 embryo (head at upper end missing); note strong ChAT in caudal corners of hindlimbs (arrows), and high expression in heart and allantois; d) by HH22, caudal ChAT expression expands rostrally; e) by HH22^+^, in both hind and front limbs ChAT has further expanded; note highest expression on caudal and distal parts of limb (cf. with f); f) for comparison, AChE expression in HH22 is concentrated rostrally; g) distinct ChAT in wing, and h) in leg of HH25 embryo; i) ChAT in ventral hind trunk of a HH25^+^ embryo. al, allantois; ht, heart; lwg, left wing; rwg, right wing; llg, left leg; rlg, right leg.

Expression of AChE is widespread in the early chicken embryo (Fig 1B; [1, 42]). Thereby, AChE delineates precisely shape of future long bones and phalanges in early limbs. In Fig 1, whole-mounted limb specimen from HH16 to HH 32 expressed pronounced activity of AChE. Already in a HH16 embryo, AChE was detectable with the earliest indication of a limb anlage along a slightly bulged ectodermal surface (Fig 1A, arrow). As the limb bud emerged, AChE covered most of it, being elevated at its rostral and towards its distal end (Fig 1C, arrows; Fig 2F; further details, see below). By HH19, the wing anlage (Fig 1C and 1D) had further extended, with an outer contour of strong AChE expression on its ventral side (Fig 1C), As shown on AChE-stained cryosection of HH19 (Fig 1D), an ectodermal layer expressed AChE, whereby a graded increase of activity to words the distal end of the limb was evident. This area corresponds with the apical ectodermal ridge (AER), the first organizing center of the vertebrate limb [43]. Notably, the ectodermal AChE staining of a HH22 cryosection was almost completely concentrated in a small group of cells at the distal end, the AER (Fig 1F). Along the limb center as a first indication of skeletal development, a bulk of stained mesenchymal cells was found amassed in proximo-distal direction, connecting with the AER. In a whole-mounted HH25 limb (Fig 1E), the outer ectodermal contour had moved more dorsally, and the inner longitudinal cell core was visible as a fine line of activity (arrow). From now onwards, the patterns became more and more distinct to outline forming skeletal structures, including digit differentiation (from Fig 1G–1I); consistently, a stronger staining towards their distal ends stood out (further see below).

#### ChAT expression is distinct and delayed, as compared with AChE

As compared with AChE in limb buds, ChAT expression was spatially distinct and temporally somewhat delayed. Whole mount embryos from stages HH16 up to HH37 were processed for ChAT expression by ISH, and were analyzed with special attention to limb development (Fig 2). ChAT expression began in the head region, while at this stage expression in limb buds was undetectable (HH17, Fig 2A). A very faint staining became discernible at HH18 on proximal bases of hind limb buds (Fig 2B, stars). Then ChAT expression extended distally; around HH21/22 ChAT became distinctively elevated at proximo-dorso-posterior corners of hind limbs (Fig 2C and 2D, arrows). Note at this time a much stronger ChAT expression in head, heart and allantois (Fig 2C). In a HH22^+^ embryo (Fig 2E), expression in the front limb had caught up with hind limb; it was strongest in distal positions (Fig 2E, arrows), whereby the interior of the limb appeared void of expression. In hind limb expression still was somewhat stronger than in front limb. Notably, while ChAT was concentrated towards postero-distally, at about the same time AChE expression was concentrated at anterior positions (cf. Fig 2F; see Discussion). From now onwards, both limb pairs elongated quickly into distal dimensions, whereby ChAT expression outlined the formation of bony structures. In Fig 2G–2I presenting details from HH25 and HH25^+^ embryos, the formation of individual bone structures became quite well detectable. A wing from HH25 (Fig 2G) presented a threefold subdivision in proximo-distal direction. In a HH25 leg (Fig 2H), two longitudinal stripes indicated fibula and tibiotarsus, and the compact staining of HH25 now announced formation of metacarpals and phalanges (cf. Fig 2H with No. 4 in Fig 2G). A ventral look on the rear part of a HH25^+^ embryo (Fig 2I) outlined further details, such as metacarpals, digits, long bones and tail structures. At later than HH25 stages, staining of exterior surfaces of limbs became much stronger, thereby blurring interior expressions of ChAT (not shown).

#### AChE is high at bone growth fronts and in degenerating tissues

**Fig 3:**
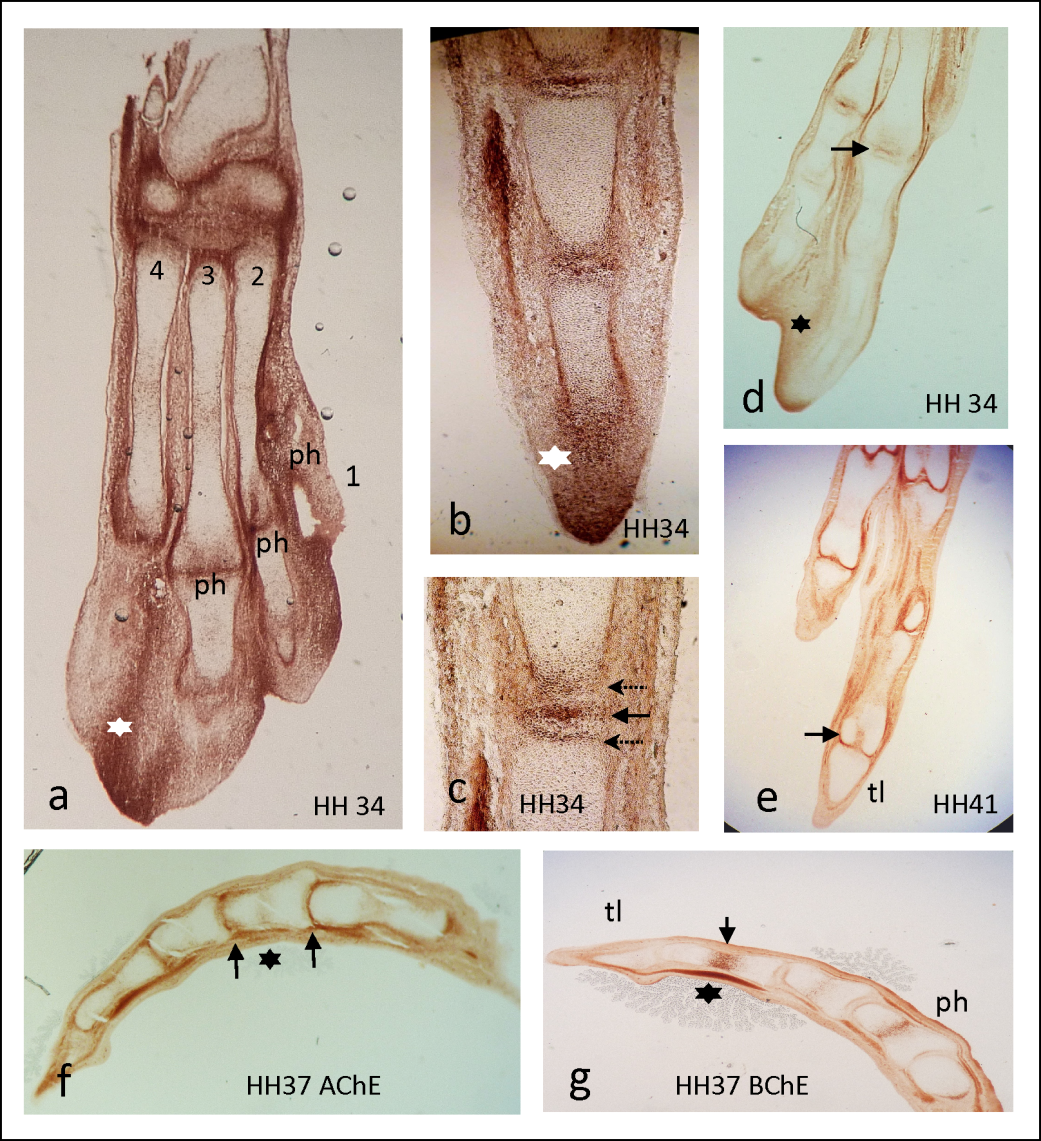
**AChE (a-f) and BChE expression (g) in maturing chicken foot.** (a) AChE in HH34 foot. Note strong expression near joints, but also in interdigital spaces (e.g., white star); metatarsi are numbered 1–4; some phalanges (ph) are visible. (b) Detail of a HH34 digit seen from side. AChE was very high at end of distal phalange and at distal tip of digits (star; cf. AER in Fig 3); (c) three-banded AChE in joint region (arrows); (d) AChE in HH34 foot viewed from side. Note ectodermal AChE at degenerating rim of interdigital space (star); (e) by HH41, digits are fully separated, AChE is very distinct in perichondria near distal end of bones (arrow); (f, g) AChE and BChE expressions in digit of a HH37 embryo; f) strong AChE at distal ends of bones, whereby most proximal bone is farthest developed, as indicated by its sharp distal border staining for AChE (right arrow); length of individual bone is indicated by two arrows. Inner end structures of bones (growth plate, epiphysis) are void of AChE, while minor staining in centres of bones (diaphysis; star) indicates bone differentiation (see also in a, e). g) BChE is strong in bone centres (arrow, g), likely associated with hematopoietic stem cells (bone marrow). Further see text; ph, phalanges; tl, talon.

As limb development proceeded, the pattern of AChE expression in feet of HH34-41 chick embryos precisely outlined the shape of maturing bones (Fig 3, also Fig 1G–1I). Fig 3A presents an overview of the entire foot structure at HH34, outlining sectioned metatarsi 2–4, and some phalanges, as well as strongly stained bone-external areas, including interdigital areas (white star in Fig 3A, see below). In a digit of a HH34 embryo (Fig 3B, detail in c), AChE staining precisely outlined shape of phalanges. Thereby, AChE activity was elevated in the future perichondrium, and most strongly in/at future joints. At higher magnification (Fig 3C) of the joint region between 1st and 2nd phalanges, a 3-banded AChE staining became evident, e.g., future perichondria at both ends of phalanges (stippled arrows in Fig 3C), and a strongly stained band in between (solid black arrow in Fig 3C). Notably, the distal end of the digit presented the highest AChE activity, where the last phalange appeared as a continuum between mesenchymal and ectodermal stained tissues (star in Fig 3B). In foot end structures of HH34 (Fig 3D), and more so by HH41 (Fig 3E) the shape of future bones had become more distinct and the pattern of AChE expression had been „clarified“, e.g. future phalanges were surrounded by a distinct and narrow perichondrial line of AChE activity. A slight staining of AChE in the center of long bones indicates onset of ossification in the diaphysis (Fig 3E and 3F). Note that in the same region BChE activity is strong (cf. Fig 3F and 3G), due to ingrowth of blood vessels.

It is well established that digit formation from young limb buds includes pronounced apoptotic cell death of tissue parts, a process that will lead to a separation of digits from each other [35]. As noted above, these interdigital areas became more and more AChE-positive, being strongest at the ectodermal rim (Fig 3A). Thereby, looking back on whole-mounted stained specimen (Fig 1G–1I) is instructive, since in whole-mounts it appeared that highest AChE activity was present at the very tips of forming digit bones, while the interdigital zones up to HH27 (stippled triangle in Fig 1G) appeared free of it. By HH30, this interdigital zone began expressing AChE (Fig 1H and 1I, stippled triangles), which had become most prominent by HH34 (Fig 3A and 3B), a time when separation of digits was ongoing. Thus, as longitudinal digit extension approached its completion, AChE expression not only outlined perichondria, future joints, particularly at distal borders of bones, but then had shifted into the interdigital zones, e.g. into apoptotic areas (see Discussion).

### 2. Loss-of-function experiments in ovo

**Fig 4:**
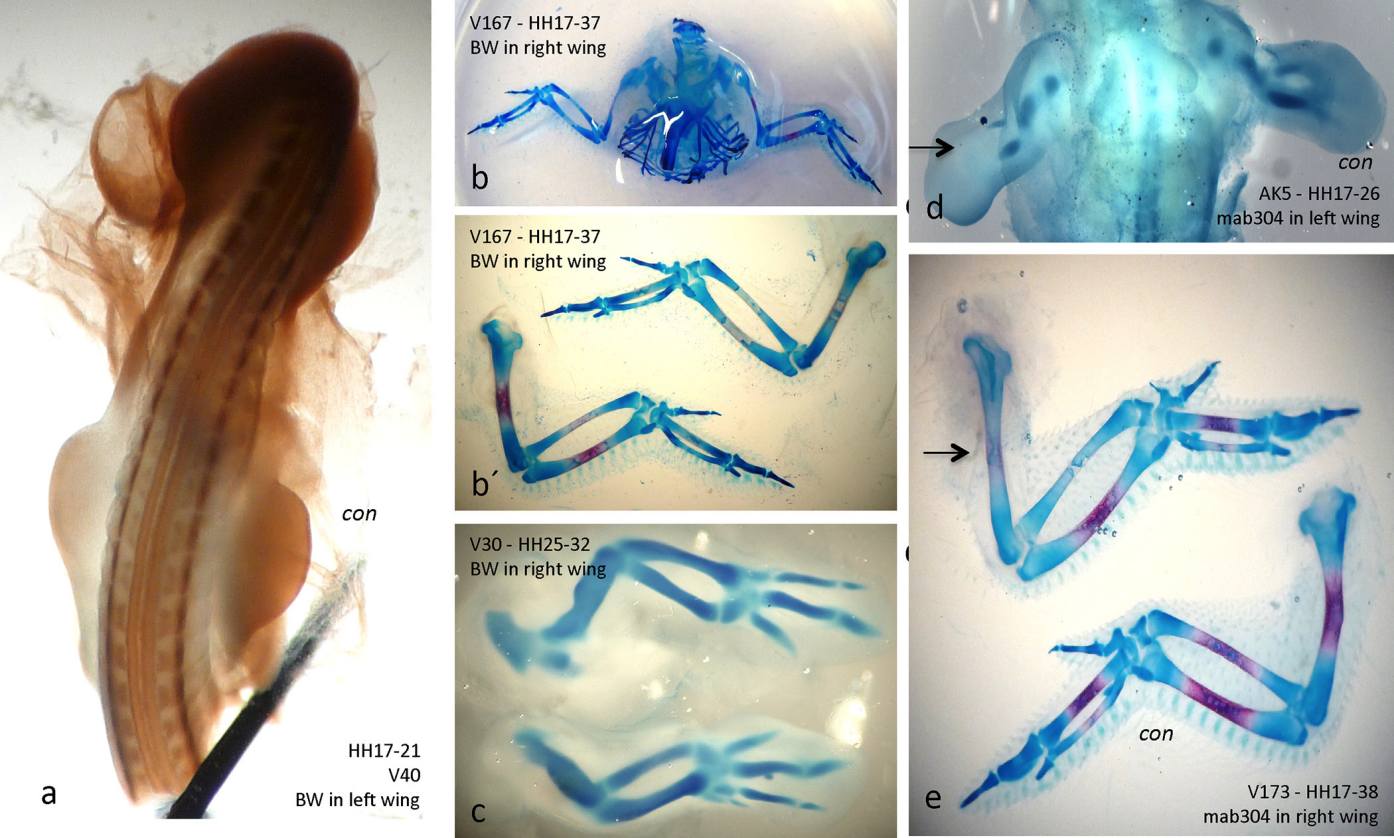
**Implantation of BW284c51- (a-c) or MAB304-soaked beads (d, e) decelerates mineralization.** a) AChE-stained HH21 embryo with a BW284c51-soaked bead implanted into left hind limb at HH17; note that at HH21 AChE activity was almost completely inhibited. b), and details in b´) BW284c51-bead implanted into right wing from HH17-37. Note severe inhibition of mineralization in treated wing (upper; blue, AB staining, red AR staining); c) BW284c51-bead implanted into right wing from HH25-32. Note smaller size of treated wing (lower; blue, AB staining). d) bead soaked in MAB304, an AChE-specific antibody, implanted into left wing (arrow) from HH17-26. Note smaller size and much retarded chondrogenesis, as revealed by AB staining (blue); e) dto, from HH17-38. Note retarded AR staining (red) in treated wing (arrow), particularly, see radius. "con", untreated control limbs.

To get a deeper insight into the functioning of the cholinergic system in chicken skeletogenesis, we have implanted beads soaked in/with ACh, ChAT, ChE inhibitors, and an AChE antibody into one limb of young chicken embryos (before HH20). The other side was left untreated and served as a control. In separate control experiments, it was established that implantation with a PBS-soaked bead did not affect limb development (for efficiency of method, see also Fig 4A). After various periods, embryos were fixed and stained with Alcian blue and/or Alizarin red to reveal chondrogenic and osteogenic structures, respectively.

#### 1. ACh-soaked beads: growth and mineralization accelerated

**Fig 5:**
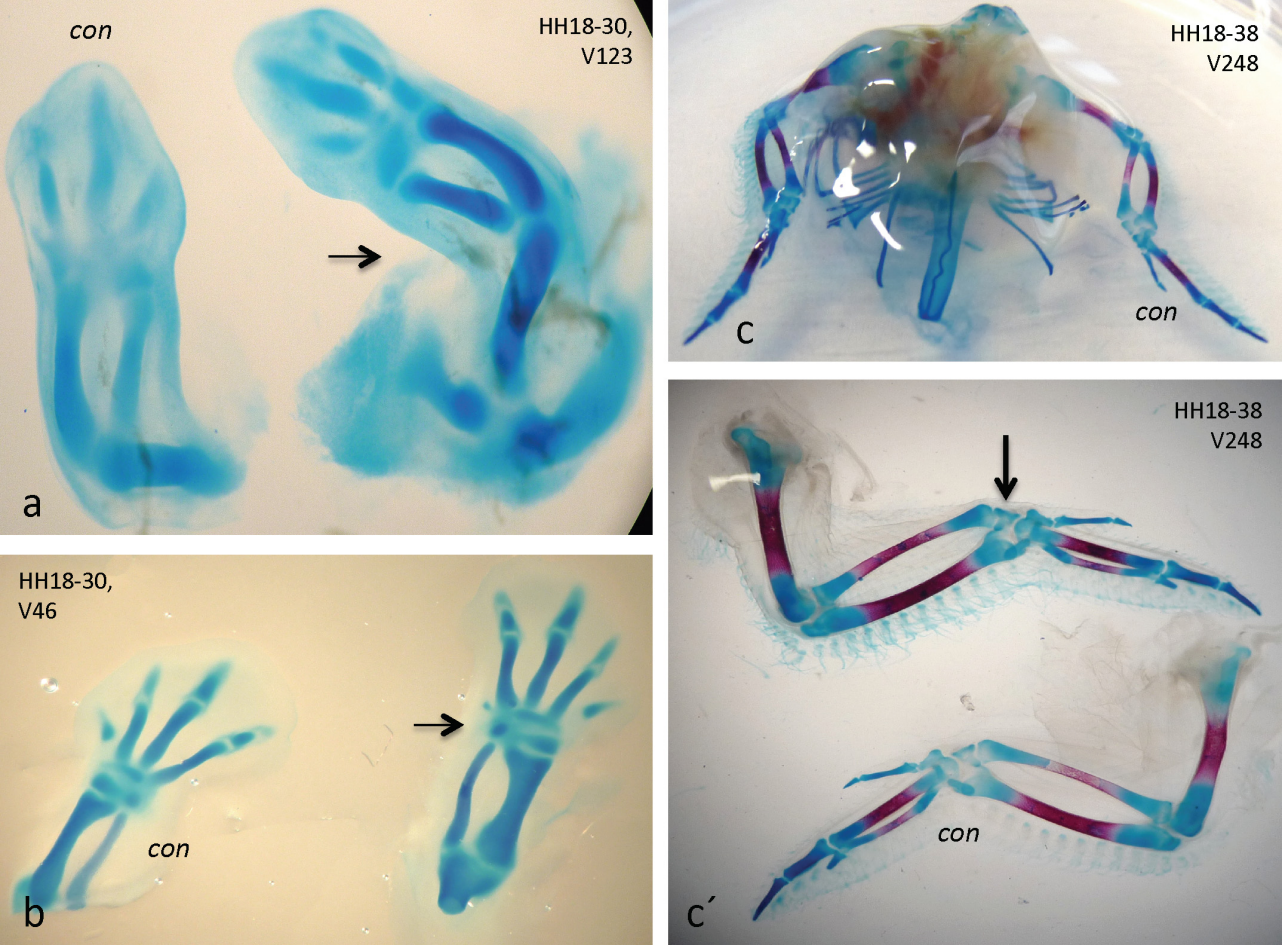
Implantation of ACh-soaked beads accelerates skeletogenesis. a) increased growth and chondrogenesis (blue, AB staining) in treated right wing (arrow) of HH18-30 embryo; b) dto. effects on treated right leg (arrow) of another HH18-30 embryo; c), details in c´) accelerated mineralization (red, AR staining) in left wing of a HH18-38 embryo. "con", untreated control limbs.

Beads were implanted into 60 embryos total, of which 48 survived (see Table 1). Thereof, 9 presented significant effects, while 39 appeared unchanged compared with control sides. In Fig 5A, a bead soaked in ACh (see Materials and Methods) had been implanted into the right wing of a HH18 embryo, which was fixed and AB stained at HH30. Clearly, growth of that wing was dramatically accelerated and its long bones (particularly, humerus, ulna and radius) were much thicker and somewhat longer than those of the control. In a separate embryo, treated equally for the same period, the effect was as evident with its treated leg (Fig 5B), best visible with a much thicker ulna. With another embryo the bead was implanted into the left wing at HH18 and only fixed at HH38 (Fig 5C, details in c´). At this late stage the differences were not as pronounced, but still a somewhat accelerated mineralization of the radius was evident. Generally, we noted that the longer the treatment period was, the weaker became its effect (see Discussion). In general, ACh in a treated limb led to accelerated growth and premature mineralization.

#### 2. ChAT-soaked beads: growth and mineralization accelerated

**Fig 6:**
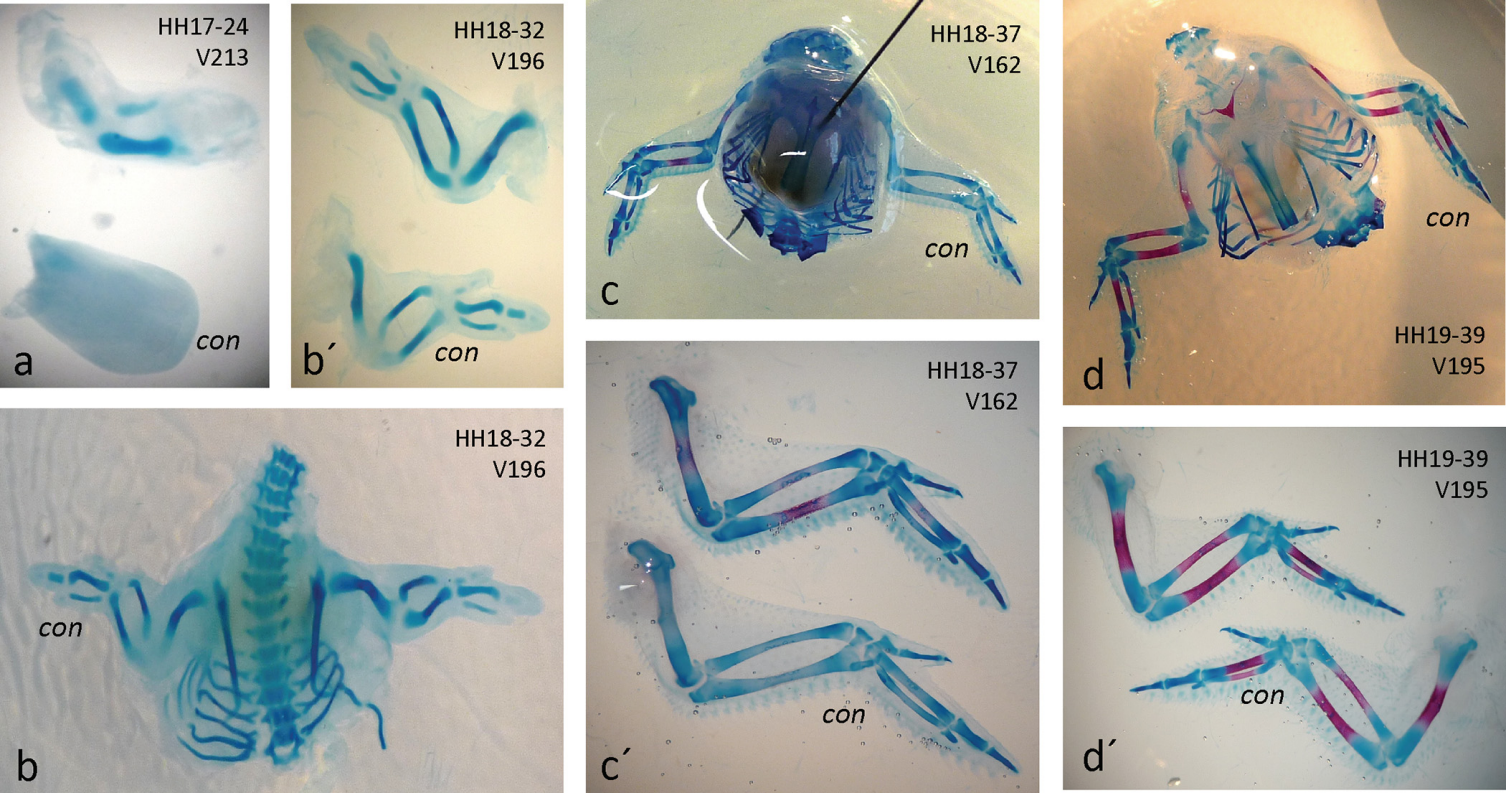
Implantation of ChAT-soaked beads accelerates skeletogenesis. a) huge developmental advance by ChAT treatment of right wing from HH17-24; b) and details in b´) accelerated growth in right wing from HH18-32; c) and details in c´) accelerated mineralization in right wing of HH18-37; d), details in d´) dto. effects in right wing of a HH19-39 embryo are slightly detectable. "con", untreated control limbs.

Implantation of beads soaked in 100 μg/ml of purified ChAT protein led to similar results as observed above with ACh beads. Of 65 embryos manipulated 46 survived, of which ten presented significant effects (Table 2, ChAT). A sample treated from HH17 to HH24 was most impressive (Fig 6A). The limb had grown much longer, and much in contrast to the control side, chondrogenesis (AB staining) of humerus, ulna and radius had clearly commenced. In a sample treated from HH18-32 a difference in limb sizes was noted (Fig 6B and 6B´), however, differences in AB staining were not as pronounced. In an HH18-HH37 treated wing mineralization was still accelerated (Fig 6C and 6C´), an effect that was also still noticeable in another embryo treated from HH19 to HH39 (Fig 6D and 6D´). In general, ChAT treatment accelerated growth and mineralization of limbs, whereby specific effects were noted at specific places (e.g. on particular digits), quite likely depending on the specific placement of the bead (see Table 2).

### 3. BW284c51-soaked beads: inhibition of AChE, but not BChE impedes ossification

Into 55 embryos total, we implanted beads soaked in BW284c51, a specific, easily reversible and bivalent inhibitor of AChE. To show the effectiveness of inhibition, a bead was implanted into the left front limb bud at stage HH17 (Fig 4A). 24 hours after implantation (by HH21) AChE activity was fully inhibited on the BW-treated (left) side, while the control side ("con" in Fig 4A) showed normal AChE activity. Such treated embryos were incubated for various periods, and then stained as whole-mounted samples for Alcian blue and Alizarin red (Fig 4B and 4C). When treated from HH17 to HH37 (Fig 4B and 4B´), quite pronounced deceleration of mineralization in long bones of the treated front limb were detectable, as compared with the control. A right wing treated from HH25 to HH32 with BW284c51 was smaller than its control (Fig 4C). In a large series of similar experiments (55 embryos treated, 47 survived; see Table 3), in 11 embryos treatment with the AChE inhibitor BW284c51 led to AChE inhibition in respected limbs, retarded growth, decelaration of chondrogenesis and mineralization and slower formation of muscle mass (see Table 3). In contrast, treatment with the BChE-specific inhibitor iso-OMPA led to conflicting results with mostly minor or no effects (not shown), which indicates that under these conditions not inhibition of ACh-degrading activities was responsible for the observed BW284c51 effects (see Discussion).

### 4. MAB304-soaked beads: periphery of AChE protein contributes to effects

Further, we applied an AChE-specific antibody, which inhibits its enzymatic activity in a complex manner (see Discussion). Treatment with the AChE-specific monoclonal MAB304 [8, 29] led to pronounced effects (Fig 4D and 4E). In a left wing sample treated from HH17-26 with MAB304, growth of the wing and its chondrogenesis were strongly inhibited (Fig 4D). A slighter effect was found in right wing when treated from HH17-38 (Fig 4E). Here, mineralization in humerus and radius were decelerated. In total, 25 embryos were treated, 21 survived, and 14 showed an effect (Table 4). For instance, in 4 out of 5 embryos, which were implanted at stage HH18, mineralization was clearly delayed (Table 4). In contrast, with Alcian blue staining (cartilage formation) only very slight effects could be noted after AChE inhibition. Noticeably, when implantation was performed only at HH22, of five embryos two had to be sorted out, and no effect was detectable in the surviving three embryos (Table 4). Since MAB304 binds in a complex manner to AChE, only partially inhibiting its enzymatic activity, these results indicate some enzymatic side and/or non-enzymatic effects of AChE preferentially on mineralization of bones during restricted time periods (see Discussion).

## Discussion

This study has provided strong support for roles of a non-neuronal cholinergic system (NNCS) in development of the chicken skeleton. Non-neuronal acetylcholine as research topic, e.g. in immune responses [44] is gaining increasing attention [5]. As a decisive component of cholinergic systems, AChE has been associated with various non-synaptic functions. Thereby, AChE´s actions can be attributed to enzymatic as well as non-enzymatic, e.g. adhesive actions (see [9, 10, 14, 22, 45]). For instance, a non-enzymatic action of AChE for *in vitro*-neurite growth has been demonstrated [7, 8]. We here have focused on limb development of the chick, since—at least during early stages—it provides an approachable non-neural developmental model in which the expression of both AChE and BChE had been described to quite some extent [27, 42], and for which knowledge of ChAT expression was meager, if not absent. This study appeared timely, after we had detected pronounced skeletal defects in perinatal embryos of double KO mice, in which both AChE and BChE were absent [9].

AChE expression presented a continuously changing pattern during limb development. Strong AChE was found consistently towards borders and tips of growing/developing structures (Figs 1 and 3), e.g. forming longitudinal subdivisions and then contours of long bones. Importantly, AChE is absent in growth plates of both chick and mouse [9, unpublished], e.g. in areas of high cell proliferation. Consistent with this, some AChE staining in long bones and phalanges was found in the center of the diaphysis, defining non-proliferative areas of differentiating hypertrophic cartilage and bone (cf. Fig 3E and 3F). Notably, AChE was found extremely high in the tip structure of the very young limb anlage, which is known as *apical ectodermal ridge* (AER; [43]), the first of two organizing centers of limb development. Thus, this indicates that AChE is involved in organizing the entire limb. In joints, AChE was expressed in a three-folded band pattern, e.g. at the two adjoining heads of long bones. In between a strong intermediate stripe of activity stood out, which could indicate an association of AChE with some ECM component. In fact, binding of AChE to collagens or laminins has been reported [3, 11, 12]. Possibly, this situation could be comparable with the association of AChE with the basal lamina of the neuromuscular junction [46].

ChAT expression has been studied in numerous neural systems ([47] for review). In lumbar spinal cord of the chick, ChAT was radiometrically detected already at E2 before final mitosis of any neuroblast [48]. Notably, a central type of ChAT (cChAT) and a peripheral ChAT (pChAT) occur through alternative splicing [49]. It is striking that—except a short notice of ChAT radiometric determination in chicken limbs [50]—only this study focused on spatial expression of ChAT during non-neuronal development. For analyzing ChAT expression, we decided to use whole-mounted specimen in order to get 3-dimensional impressions of ChAT distribution. As a corollary, the ISH stainings were not easy to resolve. Also, stainings of samples from different stages varied, which made comparisons difficult. Nevertheless, significant notions were revealed. ChAT expression follows an overall rostro-caudal gradient. Its early expression in the head region was most prominent and generally compares with AChE expression in brain and trunk [42, 51–53]. In nervous tissues, it appears that AChE is expressed slightly earlier than ChAT [47], which certainly applies for retinal development [54], and—as this study shows—most likely also for limbs. Remarkably, ChAT appeared earlier in hind than in front limb. Albeit being spatially quite similar, ChAT and AChE expression patterns did not fully overlap. While AChE was extremely high in the AER (see above), which directs the proximo-distal limb differentiation, ChAT showed an early peak in the so-called *zone of polarizing activity* (ZPA) at the posterior corner of the HH22 hind limb (Fig 2D and 2E). The ZPA is regulating the rostro-caudal and–to some degree–the dorso-ventral differentiation of limbs [43]. Often, expression of ChAT was stronger on ventral sides of the limb bud (see Fig 2C and 2I). Together, these observations strongly indicate that the synthesizing and degrading enzymes of ACh could create distinct spatial source-sink gradients of ACh within the developing limb anlage. Moreover, the two enzymes are associated with two major organizing centers of the vertebrate limb, which direct the formation of the vertebrate limb via Fgf-, Shh-, Wnt-, and BMP-related mechanisms [35]. How cholinergic effects are integrated in these processes remains a fascinating topic for future research.

### AChE in cell death

AChE has repeatedly been assigned a role in apoptotic processes [15, 23–26, 55]. In epiphysis of murine long bones, a narrow stripe of AChE^+^ cells has been detected in the apoptotic zone near the epi- to diaphysis border; a similar association of AChE with an apoptotic zone occurred in mineralizing chicken micromass cultures (Spieker et al., submitted). From images like that of Fig 3A and 3B, it appears possible that apoptotic AChE^+^ cells migrate from the bone anlage out into the mesenchymal space. The present study has demonstrated a late appearance of AChE in interdigital zones (Figs 1G–1I and 3A), as cells there go into apoptosis [43]. Notably, in AChE-stained sections of limbs from embryonic ducklings a corresponding interdigital staining was absent (Fig 1J), supporting the idea that this absence is correlated with the persistence of interdigital swimming skins in ducks. The precise molecular mechanism of how AChE affects apoptosis remains elusive, possibly AChE thereby affecting nuclear condensation and preventing caspase-9 activation [15].

### In vivo evidence for cholinergic functions in skeletogenesis

Functional analyses by *in vivo* bead implantations provided convincing evidence that skeletal development of the chicken limb depends heavily on cholinergic mechanisms. With large experimental series on living embryos (see Tables 1–4), this meticulously challenging approach allowed most revealing *in vivo* observations. The large number of treated embryos allowed us to discard all dead or injured samples, and yet we were still left with significant numbers of exploitable specimen. With this method, continuous observations and documentation from whole-mounted stained specimen became possible. A large series of embryos had been implanted with PBS-beads alone to ensure that limbs in presence of a bead were developing normally (data not shown).

Both ACh- and ChAT-soaked beads led to accelerated growth and mineralization of limbs. These two approaches directly established that bone development is regulated by an ACh-dependent mechanism. Thereby, the actions of ACh, or, of ChAT are expected to stimulate proliferation in chondroblasts and osteoblasts in the growth plate (epiphysis) of the forming bone [18–21], which represents an AChE-free space (cf. Fig 3). The degree of ACh- and ChAT-effects was variable (see Tables 1 and 2); one of the reasons for this being that local concentrations of released drugs cannot be precisely controlled. This is not only because precise placing of the bead is extremely difficult, but more so because the release of drugs, their diffusion and degradation within the tissue are not calculable. In particular this relates to ACh itself, since this small molecule will be degraded rapidly by cholinesterases present in the tissue. Thus, uncertainties in drug placement and concentration may have caused that i) patterns and degrees of defects varied, and ii) they often were less pronounced as exposure periods became longer.

### AChE also acts ACh-independently during chicken limb development

Our findings with implantation of the AChE inhibitor BW284c51 and MAB304 were perplexing at a first glance, most likely reflecting more a non-conventional functioning rather than its ACh-degrading function. Our histochemical stainings established that AChE activity in BW284c51-treated limbs indeed was blocked. Intuitively, when applying BW284c51 beads one may expect similar effects as with implanting ACh and ChAT beads, since an inhibited AChE should cause higher ACh levels in the tissue. Accordingly, it was found in another system, that both galantamine (also an AChE inhibitor) and nicotine induced the ChAT promotor and a NNCS [56]. Similarly, when we examined a cholinergic involvement in mesenchymal micromass cultures from chick limb buds, treatments with both nicotine or with BW284c51 of cartilage-like nodules accelerated mineralization, an effect that was at least partially mediated via the α7-nAChR (Spieker et al., submitted). With the present *in vivo* study, which is entirely different from micromass cultures, however, BW284c51 delayed mineralization. To interpret these striking BW284c51 findings, temporal and spatial features of cell proliferation and differentiation in relation to AChE expression in long bones need consideration. Cell proliferation takes mainly place in the growth plates (epiphysis), e.g. at both ends of a forming long bone ([43], cf. Fig 3E and 3F) at a time when there is yet no AChE within the forming bone tissue. Dependent on Runx2 activity [22], and only with onset of mineralization, AChE will be elevated in the diaphysis (see Fig 3F). Hence, inhibition of AChE is not expected to affect cell proliferation. Being restricted to the mineralization zone, AChE could then subserve other ACh-dependent, or ACh-independent function(s).

Remarkably, our findings with BW284c51 inhibition were similar to those with MAB304. BW284c51 is a bivalent ligand binding to a central (CAS) and a peripheral anionic binding site (PAS) of AChE. MAB304 binding on AChE protein and its effects are rather complex [34, 35]. For instance, MAB304 inhibits AChE activity to a large degree, while not interfering with a series of central and/or peripheral ligands [34, 35]. Notably similar to BW284c51, MAB304 binds to CAS [33]. MAB304 recognizes the motif YQYVD [29], whereby D of this sequence corresponds to Asp74 of AChE, which constitutes part of a side door in the crystal structure of AChE [57]. These features of ligand selectivities and binding topology of MAB304 were reminiscent of a so-called aryl acylamidase (AAA) activity associated with the AChE protein [28], and suggest AAA as a possible target for MAB304. This side door has been implicated in AAA activity, possibly using it for trafficking [30]. AAA on AChE is elevated during early developmental periods in the chick [28], it is more sensitive to BW284c51 inhibition than is AChE activity itself [28], and—most significantly—has been found to promote differentiation of an osteoblastic cell line (pers. communication Dr. R. Boopathy and Dr. C. Rajkumar). Taken together, these features suggest that this antibody will block the area of the side door together with AAA activity. Therefore both, MAB304 and BW284c51 could have achieved their effects on bone development by similar, partially ACh-independent actions during the mineralization period of long bones, whereby inhibition of AAA could be involved. This interpretation is strongly supported by our extensive bead inhibitions of BChE, which only slightly, if not at all affected limb formation.

## Conclusions

During early limb development, AChE is strongly expressed at differentiation centers and growth borders of forming bones and perichondria, and eventually is elevated in apoptotic areas. In limb buds, AChE appears shortly before ChAT. A plethora of studies strongly supports cholinergic functions in chondro- and osteoblasts, however, most of them were in vitro studies. Our present bead implantation experiments allowed direct testing of cholinergic effects on avian limb development. ACh and ChAT stimulated skeletogenic events in limbs. In addition, AChE inhibition was shown to affect bone development—at least partially—via an ACh-independent mechanism. These findings have significant relevance for understanding genetically or environmentally caused skeletal diseases. This particular topic of NNCS asks for much more research.

## Abbreviations

AB: Alcian blue
ACh: acetylcholine
AChE: acetylcholinesterase
AR: Alizarin red
AER: apical ectodermal ridge
BChE: butyrylcholinesterase
BW284c51: 1,5-bis-(4-allyldimethylammoniumphenyl)pentan-3-one dibromide, a specific inhibitor of AChE
ChAT: choline acetyltransferase
ISH: in situ hybridization
iso-OMPA: tetra-isopropyl-pyrophosphoramide, a specific inhibitor of BChE
ZPA: zone of polarizing activity
